# Intraspinal bone-marrow cell therapy at pre- and symptomatic phases in a mouse model of amyotrophic lateral sclerosis

**DOI:** 10.1186/s13287-016-0293-4

**Published:** 2016-03-15

**Authors:** Fernanda Gubert, Ana B. Decotelli, Igor Bonacossa-Pereira, Fernanda R. Figueiredo, Camila Zaverucha-do-Valle, Fernanda Tovar-Moll, Luísa Hoffmann, Turan P. Urmenyi, Marcelo F. Santiago, Rosalia Mendez-Otero

**Affiliations:** Instituto de Biofísica Carlos Chagas Filho, Centro de Ciências da Saúde, Sala G2-028, Universidade Federal do Rio de Janeiro, Cidade Universitária, RJ 21941-902 Rio de Janeiro, Brazil; Evandro Chagas National Institute of Infectious Diseases (INI), Oswaldo Cruz Foundation, Avenida Brasil 4365, Maguinhos RJ 21040-900 Rio de Janeiro, Brazil; Institute of Biomedical Sciences and National Center of Structural Biology and Bioimaging, CENABIO, Centro de Ciências da Saúde, Universidade Federal do Rio de Janeiro, Cidade Universitária, RJ 21941-902 Rio de Janeiro, Brazil; Instituto D’Or de Pesquisa e Educação (IDOR), Rua Diniz Cordeiro 30, Botafogo RJ 22281-100 Rio de Janeiro, Brazil

**Keywords:** Amyotrophic lateral sclerosis, Bone marrow cell therapy, Neuronal degeneration

## Abstract

**Background:**

Amyotrophic lateral sclerosis (ALS) is a progressive neurological disease that selectively affects the motor neurons. The details of the mechanisms of selective motor-neuron death remain unknown and no effective therapy has been developed. We investigated the therapy with bone-marrow mononuclear cells (BMMC) in a mouse model of ALS (SOD1^G93A^ mice).

**Methods:**

We injected 10^6^ BMMC into the lumbar portion of the spinal cord of SOD1^G93A^ mice in presymptomatic (9 weeks old) and symptomatic (14 weeks old) phases. In each condition, we analyzed the progression of disease and the lifespan of the animals.

**Results:**

We observed a mild transitory delay in the disease progression in the animals injected with BMMC in the presymptomatic phase. However, we observed no increase in the lifespan. When we injected BMMC in the symptomatic phase, we observed no difference in the animals’ lifespan or in the disease progression. Immunohistochemistry for NeuN showed a decrease in the number of motor neurons during the course of the disease, and this decrease was not affected by either treatment. Using different strategies to track the BMMC, we noted that few cells remained in the spinal cord after transplantation. This observation could explain why the BMMC therapy had only a transitory effect.

**Conclusion:**

This is the first report of intraspinal BMMC therapy in a mouse model of ALS. We conclude this cellular therapy has only a mild transitory effect when performed in the presymptomatic phase of the disease.

**Electronic supplementary material:**

The online version of this article (doi:10.1186/s13287-016-0293-4) contains supplementary material, which is available to authorized users.

## Background

Amyotrophic lateral sclerosis (ALS) is a fatal neurodegenerative disease involving the death of motor neurons in the spinal cord, brain stem, and motor cortex. Approximately 90 % of ALS cases are sporadic, and the remaining 10 % are inherited (familial). A mutation in the Cu/Zn superoxide dismutase 1 (SOD1) gene is responsible for 20 % of the familial forms [1]. In rodents, overexpression of the human SOD1 mutant protein leads to progressive motor-neuron degeneration, which mimics the pathological progression observed in humans [2, 3].

Several lines of evidence suggest that nonneuronal cells play an important role in ALS pathogenesis, since both the astrocytes and the microglia expressing the mutated SOD1 are toxic to motor neurons and contribute significantly to the disease progression [4–8]. In addition, immune cells such as T lymphocytes have also been implicated in ALS, and an increase in the number of these cells in the affected areas of the central nervous system (CNS) has been described both in humans and in mouse models [9–11]. The CNS-infiltrating CD4 T lymphocytes seem to communicate with the microglia, and this dialog directs the microglia to a neuroprotective profile [12, 13].

Currently, the only treatment for ALS is Riluzole®, Sanofi S.A., (Gentilly, Paris, France), which increases the patient’s lifespan by only 3 months [14]. Alternatively, cell therapies with different types of stem cells have been suggested as a possible therapeutic approach. Bone marrow-derived stem cells have been used in several models of neurological disease, including ALS [15–19]. In several of these studies, beneficial effects were observed; the suggested mechanism of action includes release of trophic factors and local or systemic modulation of the immune response.

In addition to different cell types, different routes of injection have been tested in animal ALS models; for instance, intravenous, intrathecal, intramuscular, and direct delivery in the spinal cord [16, 20–22]. In the majority of studies using animal ALS models, the cells were injected in the presymptomatic phase of the disease. For clinical translation, however, cell therapy during the symptomatic phase could be more meaningful. In this respect, a few clinical trials with bone marrow mononuclear cells or mesenchymal stem cells in ALS patients showed that these therapies are safe, although the efficacy of these therapies still needs to be investigated [23, 24].

In this study, we investigated the potential therapeutic benefit of bone marrow-derived mononuclear cells (BMMC) injected directly into the lumbar spinal cord of ALS mice in the presymptomatic or symptomatic phases. BMMC have been broadly tested in preclinical models of traumas or neurodegenerative diseases in the nervous system, showing positive results. These cells are easy to isolate and require minimum manipulation prior to transplant, becoming a good candidate for cell therapies. Using different models of neurological diseases, our group showed in previous publications that BMMC transplantation results in neuronal protection, axonal regeneration, decreases microglia activation, and improves functional recovery [17, 25, 26]. More importantly, this is the first study using this approach, and we report here that the BMMC therapy delayed the animals’ functional outcome only when administered in the presymptomatic phase.

## Methods

### Animals

All of the experiments were conducted in accordance with the recommendations of the National Institutes of Health Guide for the Care and Use of Laboratory Animals, and were approved by the Committee for the Use of Experimental Animals of our institution (permit number: IBCCF213-09/16). The strain of mice used was B6SJL-Tg(SOD1-G93A)1Gur (SOD1^G93A^), developed by Gurney in 1994 [2], which carries the mutant allele human SOD1 containing the Gly 93 → Ala substitution. The colony was maintained by crossing transgenic male founders with wild-type female mice. The number of human SOD1 transgenic copies was assessed as described in the Jackson Laboratory manual (https://www.jax.org/strain/002726#jump-nav-5). The breeding pairs were donated by the ALS Foundation through Dr R Brown (University of Massachusetts, Worcester, USA).

### Isolation and labeling of mononuclear bone marrow cells

Bone marrow was obtained from the femur and tibia of adult B6SJL mice of both genders. BMMC were isolated by density gradient (Histopaque 1083; Sigma, St. Louis, MO, USA) and washed three times with phosphate-buffered saline (PBS). Cells were suspended in saline (250,000 cells/μl) and injected into the lumbar portion of the spinal cord as described in the next section.

In some animals the BMMC were labeled, before transplantation, with a fluorescent marker (CellTrace™ Far Red DDAO-SE; Life Technologies, Carlsbad, CA, USA) or with iron nanoparticles (superparamagnetic iron nanoparticles (SPION), FeraTrack™; Miltenyi Biotec, Bergisch Gladbach, Germany). For the fluorescent labeling, BMMC were incubated with Cell Trace™ (1:1000) diluted in Dulbecco’s modified Eagle’s medium (DMEM)–F12 (Life Technologies) for 40 minutes at 37 ° C, washed five times with PBS, and then suspended in saline.

For the SPION labeling, BMMC were incubated with FeraTrack™ contrast particles and FeraTrack™ loading reagent for 3 hours at 37 °C/5 % CO_2_, as described previously by Zaverucha-do-Valle et al. [27]. After incubation, the cells were washed three times with PBS and suspended in saline. The labeled cells were injected in week 9 or week 14. Cells were tracked in vivo by magnetic resonance imaging (MRI) measurements at 1 and 7 days after the injection and in the end stage of the disease.

Images were acquired with a 7 T magnetic resonance scanner (7 T/400 horizontal Varian scanner; Agilent Technologies, Santa Clara, CA, USA). Four proton-density fast spin-echo imaging sequences (repetition time = 2000 milliseconds; echo spacing = 10.5–12 milliseconds: matrix = 192 × 192; slice thickness = 0.5 mm; 7–10 continuous slices, no gap; 16 averages, axial field of view (FOV) = 25 mm × 25 mm, coronal FOV = 30 mm × 30 mm, sagittal FOV = 40 mm × 30 mm, total sagittal FOV = 50 mm × 30 mm) were used to investigate the spinal cord region.

Prior to image analysis, datasets were anonymized. For each dataset, all images were visually inspected for important artifacts. Data were processed using MRIcron software (Columbia, SC, USA).

### Injection

SOD1^G93A^ animals were injected with BMMC or vehicle (saline) at 9 or 14 weeks old. Mice were anesthetized with xylazine (15 mg/kg; Vetbrands and ketamine (150 mg/kg; Vetbrands) (Goiânia, GO, Brazil) intraperitoneally. The animals were immobilized and the spine was exposed. The vertebrae were carefully separated using two fine tweezers in order to reveal the lumbar spinal cord (L4–L5). The BMMC (10^6^ cells) or saline were injected intraparenchymally with a glass micropipette connected to the nanoinjector (Nanoinject II; Drummond Scientific Company, Broomall, PA, USA) at the rate of 1 μl/minute for a total volume of 4 μl. After recovery from anesthesia, the animals from both groups were returned to the animal facility and kept in cages with food and water ad libitum*.* To assess the survival and functional outcome, the animals were divided into five groups: BMMC treated in week 9 (*n* = 22), saline injected in week 9 (*n* = 24), BMMC treated in week 14 (*n* = 22), saline injected in week 14 (*n* = 22), and wild-type (*n* = 16). Equal numbers of male and female were used in each group.

### Functional evaluation

Functional tests were performed weekly in all experimental groups by blinded investigators. The tests performed were the rotarod, hanging wire, and motor score. In the rotarod test, the animals were placed in a rolling rod (maximum of 180 seconds) with an accelerating speed from 20 to 35 rpm. In the hanging-wire test, the animals were placed on the wire lid from their housing cage, where they remained upside down until they fell (maximum time 90 seconds). The longest period that the animal remained in the rotarod test and hanging-wire test was recorded after three trials.

In the motor score, the animals were graded as follows: 4 = no sign of motor dysfunction, 3 = hind-limb tremors when suspended by the tail and collapse or partial collapse of leg extension, 2 = evident motor dysfunction, 1 = paralysis in at least one limb, and 0 = not able to right itself within 30 seconds when placed on its side.

The statistical analyses were performed with the two-way analysis of variance (ANOVA) and Bonferroni post-test in GraphPad Prism 4.02 (GraphPad Software, Inc., La Jolla, CA, USA). For survival comparison, the log-rank test was used. Statistical significance was considered when *p* ≤0.05. All data are presented as mean ± standard error of the mean (SEM).

### Tissue preparation and immunohistochemistry

Animals (*n* = 5/6 animals per group) were perfused through the heart with ice-cold saline, followed by a solution of 4 % paraformaldehyde in 0.1 M phosphate buffer, pH 7.4, via the ascending aorta. The spinal cords were then removed, and cryoprotected by immersion in 20 % sucrose followed by 30 % sucrose in phosphate buffer. The spinal cords were sectioned transversely at 20 μm on a cryostat (CM 1850; Leica, Wetzlar, Germany) and collected on gelatin-coated slides.

For immunohistochemistry, sections were washed three times with PBS with 0.3 % Triton X-100, incubated with 5 % normal goat serum (NGS; Sigma) in PBS for 30 minutes, and then incubated with the primary antibodies monoclonal mouse anti-NeuN (1:200, #MAB 377; Chemicon, Temecula, CA, USA) and polyclonal rabbit anti-Iba1 (1:400, #019-19741; Wako, Richmond, VA, USA) overnight at 4 °C. After the primary incubations, the appropriate secondary Cy3®-conjugated or Alexa® 488-conjugated antibodies (Jackson ImmunoResearch, West Grove, PA, USA and Invitrogen Inc., Carlsbad, CA, respectively) were used. In some cases, the sections were incubated with TO-PRO®-3 (1:1000; Invitrogen) for nuclei staining. Sections were mounted with VectaShield® (Vector, Burlingame, CA, USA) and analyzed using confocal microscopes (LSM 510 META or LSM 510 META NLO; Zeiss GmbH, Oberkochen, Germany).

### Motor-neuron and microglia quantification

For motor-neuron quantification we stained the lumbar spinal cord with NeuN antibody. We inspected every fifth transverse section of a total of 10 sections from the anterior horn of the lumbar spinal cord (20 μm). Counts were made using a 20× objective on a Zeiss Axiovert 200 M microscope equipped with fluorescence optics. The size of NeuN-positive cells in the anterior horn was quantified; cells featuring cell bodies with cross-sectional area ≥250 μm^2^ were considered motor neurons. Smaller NeuN-positive cells were considered interneurons.

For microglia quantification, we stained the spinal cord with Iba1 antibody. We quantified every fifth section of a total of 20 transverse sections from the anterior horn of the lumbar spinal cord (20 μm). Counts were made using a 40× objective on a Zeiss Axiovert 200 M microscope with fluorescence optics.

Statistical analysis was performed by one-way ANOVA and Tukey’s multiple comparison test in GraphPad Prism 4.02 (GraphPad Software, Inc., La Jolla, CA, USA). Statistical significance was considered when *p* ≤0.05. All data are presented as mean ± SEM.

## Results

### Effect of BMMC treatment on motor functional outcome

To assess the effect of BMMC transplanted directly into the lumbar spinal cord, we analyzed the disease functional outcome using the rotarod, hanging-wire, and motor-score tests. We divided the animals into two groups: presymptomatic animals, which were injected with saline (*n* = 24) or BMMC (*n* = 22) in week 9; and symptomatic animals, which received the injection only in week 14 (saline, *n* = 22; BMMC, *n* = 22). These groups were also compared with wild-type animals of equivalent ages (*n* = 16).

**Fig. 1:**
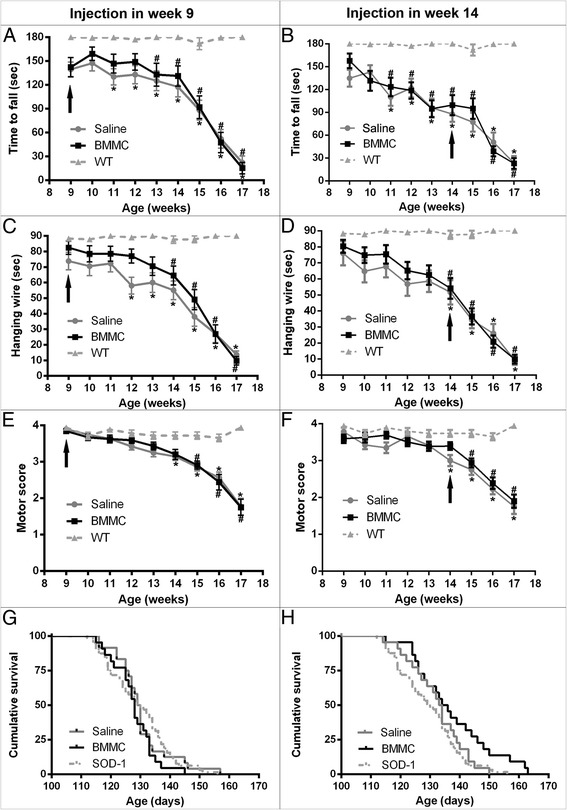
Functional outcome and survival of SOD-1^G93A^ mice after BMMC therapy. **a, b** Rotarod test, **c, d** hanging-wire test, **e, f** motor-score test, and **g, h** cumulative survival. SOD-1^G93A^ mice were injected with BMMCs or saline at two different time points: presymptomatic phase (9 weeks old **a, c, e, g**) and symptomatic phase (14 weeks old **b, d, f, h**). The animals that received the treatment in week 9 showed a 2-week delay in the functional loss in the rotarod and hanging-wire tests. In the motor-score analyses, the saline-injected animals in week 9 showed a decline in week 14, while the BMMC-treated animals showed a decline in week 15. No differences were observed between the saline-injected and BMMC-injected animals in week 9 in the cumulative survival. The therapy in week 14 did not show an effect in any analysis performed. *Arrows* indicate week of treatment. *WT vs. saline (*p* <0.05), #WT vs. BMMC (*p* <0.05). *BMMC* bone marrow-derived mononuclear cells, *SOD1* Cu/Zn superoxide dismutase 1, *WT* wild-type

In the rotarod test, compared with the wild-type mice, the animals treated with BMMC in the presymptomatic phase started to show motor deficits in week 13, while in the untreated group this decline was already apparent in week 11 (Fig. 1a). In the hanging-wire test, we also observed that the saline group started to show loss of function 2 weeks before the treated group (Fig. 1c). In the motor score analyses, we observed a delay in the functional loss in the BMMC-treated animals in week 9 (Fig. 1e). This group showed deficits in week 15 compared with wild-type mice, while saline injected animals showed a deficit already in week 14. However, we did not observe differences between the BMMC-injected and saline-injected animals.

The animals treated with BMMCs in the symptomatic phase showed motor deficit as early as at the injection day when measured by all functional tests; and these deficits were never recovered or stabilized (Fig. 1b, d, f).

**Fig. 2:**
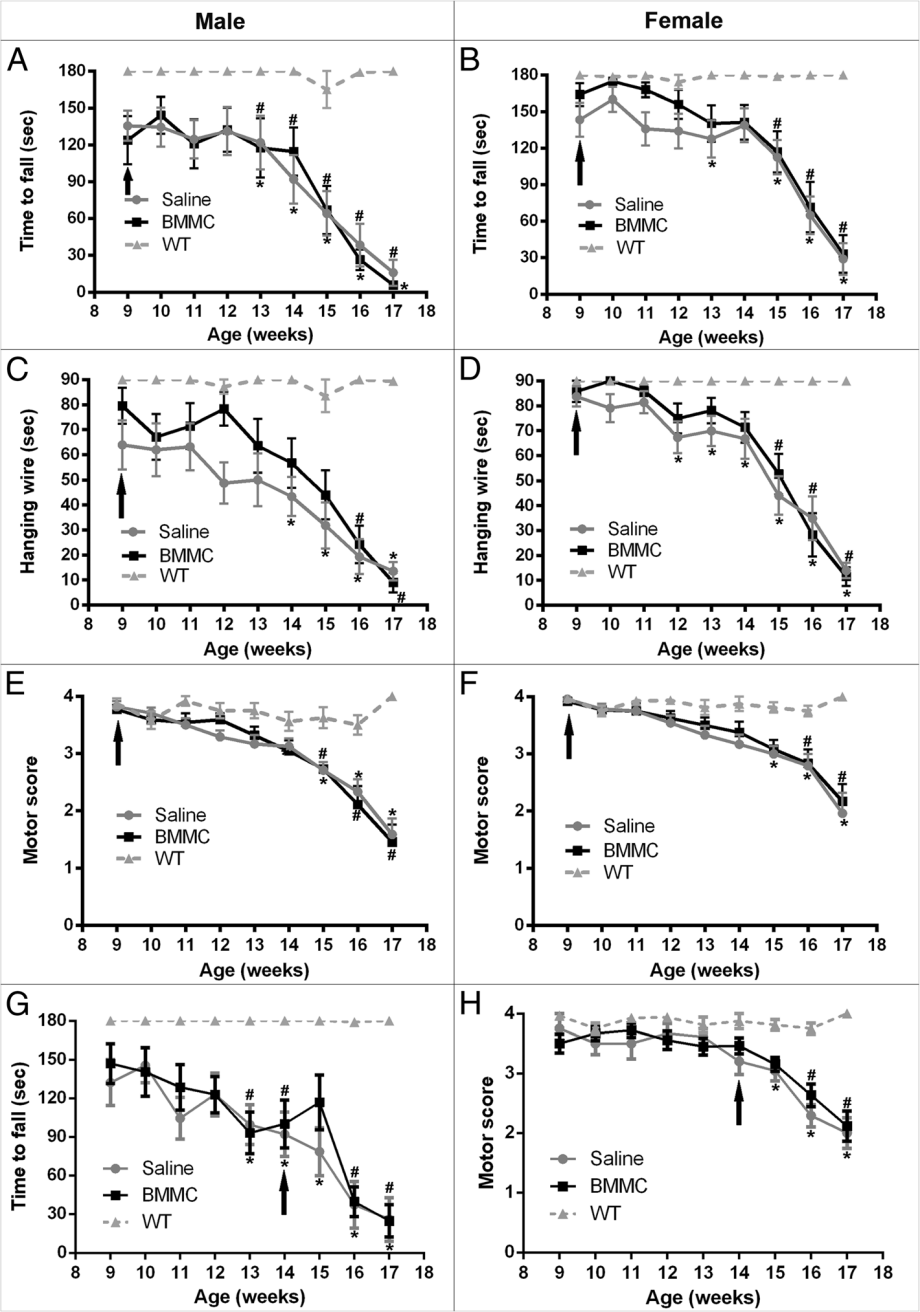
Gender effect in the functional outcome of SOD-1^G93A^ mice after BMMC therapy. **a, b, g** Rotarod test, **c, d** hanging-wire test, and **e, f, h** motor-score test. Female SOD-1^G93A^ animals that received the treatment in week 9 showed a delay in the functional loss in all tests performed **b, d, f**. Male animals treated in week 9 showed a delay only in the hanging-wire test **c**. The therapy in week 14 improved the rotarod performance in males **g** and delayed the deficit analyzed by motor score grade in BMMC-injected females **h**. *Arrows* indicate week of treatment. *WT vs. saline (*p* <0.05), #WT vs. BMMC (*p* <0.05). *BMMC* bone marrow-derived mononuclear cells, *WT* wild-type

To determine whether the treatment could affect differently males and females, we performed a new analysis by gender. In the presymptomatic animals, we could observe that female mice treated with BMMC showed a better performance in all tests described than the saline-injected female mice, when we compared both of them with the wild-type group (Fig. 2b, d, f). Male animals treated in week 9 showed a difference only in the hanging-wire test, where we could observe a delay in the functional loss (Fig. 2c). In the symptomatic animals, we observed a difference in the treated male animals only for the rotarod test (Fig. 2g). In this test, we observed that 1 week after treatment the animals improved their performance (week 15; Fig. 2g). However, this effect was not sustained in the following weeks. Moreover, when we compared both female groups with wild-type females we observed a 1-week delay in the manifestation of motor-score deficits in the BMMC animals when compared with the saline group (Fig. 2h).

There were no differences in survival between the BMMC-treated animals in the presymptomatic phase or the BMMC-treated animals in the symptomatic phase compared with the untreated controls (BMMC presymptomatic: 127.86 ± 7.05 days, saline presymptomatic: 130.33 ± 9.12 days, *p* = 0.303; BMMC postsymptomatic: 137.27 ± 13 days, saline postsymptomatic: 132.45 ± 9.323 days, *p* = 0.11) (Fig. 1g, h). However, we observed an increase in survival of BMMC-treated animals, in week 14, compared with noninjected mice (SOD1 noninjected: 129.54 ± 10.4; *n* = 64; male:female = 1:1; *p* <0.05) (Fig. 1h). Also, it is important to notice that only BMMC-treated animals in the symptomatic phase (two of a total of 22; male:female = 1:1) survived longer than 160 days. We did not observe any gender effect in the survival in animals treated in week 9 (male BMMC presymptomatic: 125.90 ± 7.84 days, male saline presymptomatic: 130.46 ± 7.81 days, *p* = 0.06; female BMMC presymptomatic: 130.07 ± 6.43 days, female saline presymptomatic: 130 ± 10.38 days, *p* = 0.92). We also did not observe a difference by gender in the animals treated in week 14 (male BMMC postsymptomatic: 134.45 ± 14.55 days, male saline postsymptomatic: 129 ± 9.90 days, *p* = 0.35; female BMMC postsymptomatic: 138.53 ± 10.98 days, female saline postsymptomatic: 134.58 ± 8.4 days, *p* = 0.33).

### Neuronal survival and microglia activation

**Fig. 3:**
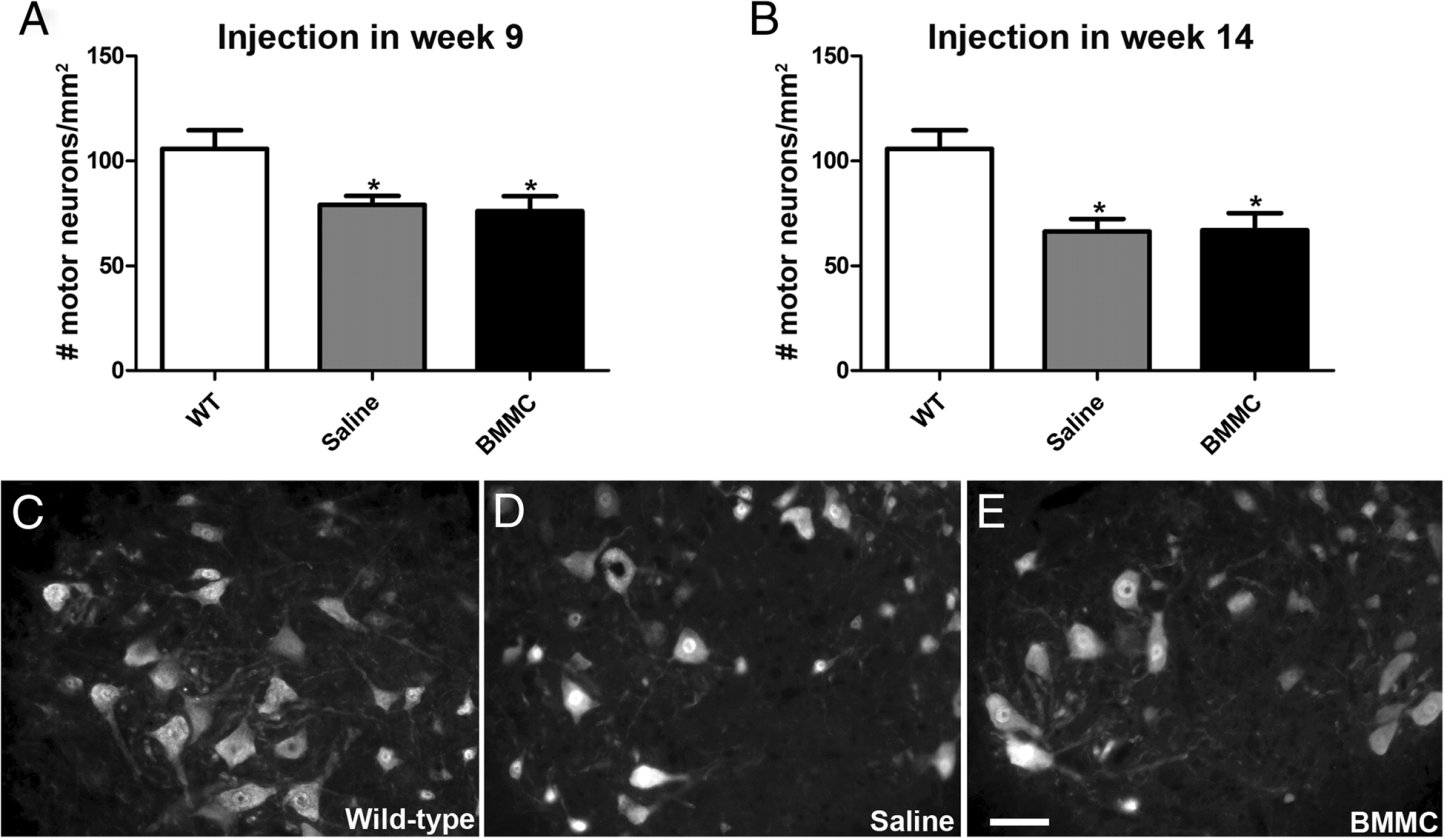
Quantification of motor neurons in the anterior horn of the spinal cord. The number of NeuN-positive cells was quantified in week 12 in the presymptomatic injected animals **a** and in week 15 in the symptomatic injected animals **b**. Both saline-injected and BMMC-injected mice showed a decrease in the number of motor neurons compared with the wild-type mice at the time point analyzed. There was no difference in the number of motor neurons after the treatment with BMMC injected in week 9 or in week 14 **c–e**. Representative images of NeuN expression in wild-type mice **c**, saline-injected animals in week 9 **d**, and BMMC-injected animals in week 9 **e**. *p <0.01. Scale bar: 50 μm. *BMMC* bone marrow-derived mononuclear cells, *WT* wild-type

To determine whether the BMMC injection could have any local effect on the spinal cord, we analyzed the neuronal survival and microglia activation in the anterior horn of the lumbar spinal cord. Neuronal survival was analyzed using NeuN staining, and motor neurons were distinguished from interneurons by size (see Materials and methods; Fig. 3). We analyzed the spinal cord of the animals injected in the presymptomatic phase in week 12, 3 weeks after the injection. At this age we could already observe a significant decrease in the number of motor neurons in the lumbar spinal cord compared with the wild-type animals. These data are consistent with the motor-function analyses, which showed a decline in the untreated animals in week 12. The BMMC treatment in the presymptomatic phase did not affect the motor-neuron survival (saline injected in week 9: 79.02 ± 11.3 cells/mm^2^, *n* = 6; BMMC injected in week 9: 76.05 ± 17.54 cells/mm^2^, *n* = 5; wild-type: 105.8 ± 19.75 cells/mm^2^, *n* = 4) (Fig. 3a).

In the symptomatic treatment protocol, we analyzed the spinal cord in week 15, 1 week after the BMMC or saline injection. As expected, we observed a significant reduction in the number of motor neurons in the untreated animals compared with the wild-type; however, the treatment did not prevent this loss (saline injected in week 14: 66.3 ± 14.69 cells/mm^2^, *n* = 7; BMMC injected in week 14: 66.96 ± 23.04 cells/mm^2^, *n* = 8) (Fig. 3b). Importantly, the number of interneurons observed in the anterior horn did not change in the transgenic animals compared with the wild-type animals at the time points analyzed (saline injected in week 9: 132.8 ± 74.64 cells/mm^2^, *n* = 6; BMMC injected in week 9: 167.3 ± 87.53 cells/mm^2^, *n* = 6; saline injected in week 14: 159.3 ± 73.61 cells/mm^2,^*n* = 8; BMMC injected in week 14: 142.2 ± 41.99 cells/mm^2^, *n* = 8; wild-type: 168.6 ± 102.7 cells/mm^2^, *n* = 7) (Additional file 1: Figure S1). At the end stage of the disease, we observed a more extensive loss of motor neurons in the anterior horn. In this case we also did not observe any preservation of motor neurons after the BMMC injection, in either the presymptomatic or symptomatic phase (saline injected in week 9: 24.03 ± 6.33 cells/mm^2^, *n* = 4; BMMC injected in week 9: 25.63 ± 14.9 cells/mm^2^, *n* = 4; saline injected in week 14: 26.88 ± 10.92 cells/mm^2^, *n* = 5; BMMC injected in week 14: 14.68 ± 11.66 cells/mm^2^, *n* = 5) (Additional file 1: Figure S1).

**Fig. 4:**
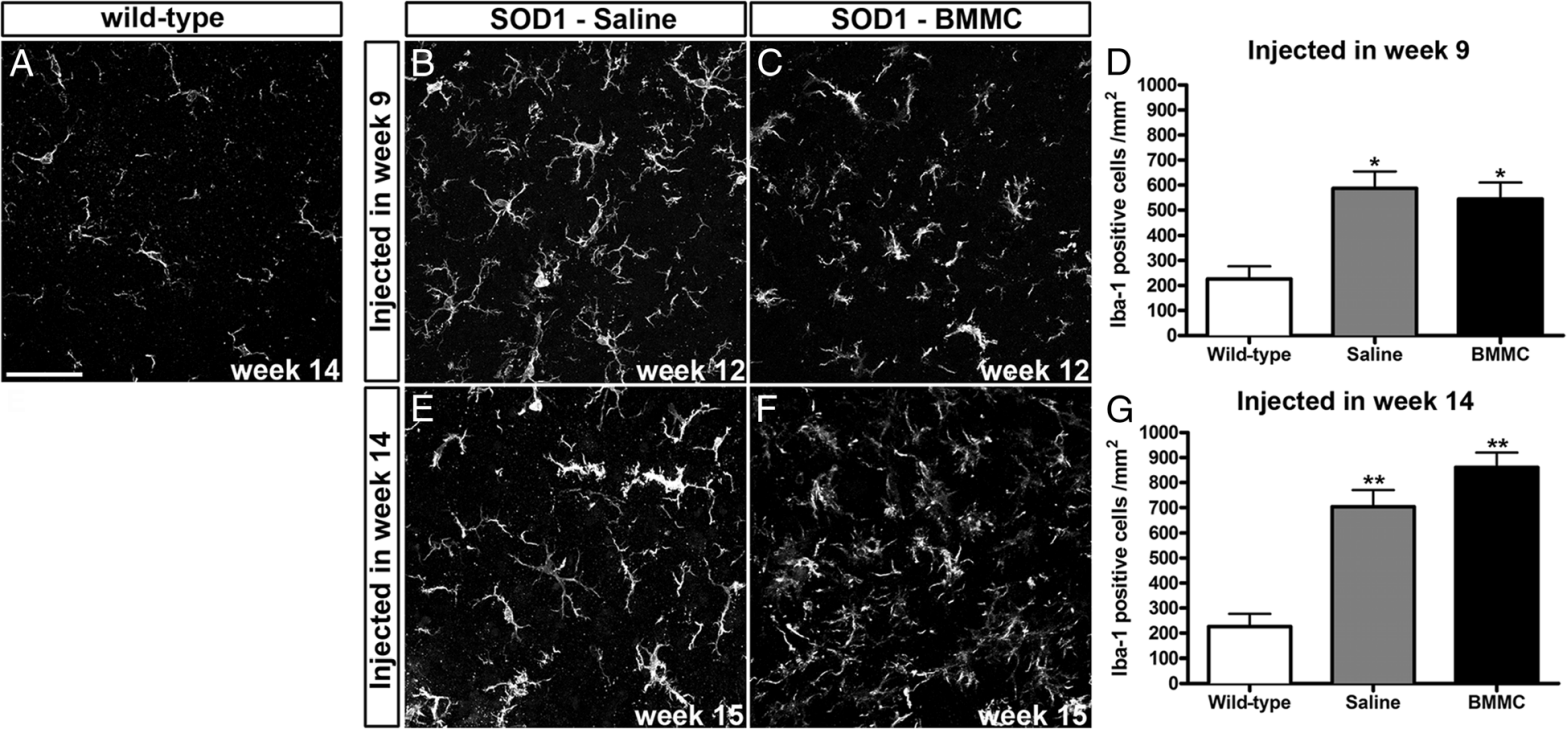
Quantification of microglial cells in the anterior horn of the spinal cord. The number of Iba1-positive cells was quantified in week 12 in the presymptomatic injected animals **b–d** and in week 15 in the symptomatic injected animals **e–g**. Iba1-positive cells in the wild-type animals are shown in **a**. The saline-injected and BMMC-injected mice showed an increase in the number of Iba1-positive cells compared with the wild-type mice. There was no difference in the number of microglia cells after the treatment with BMMC injected in week 9 or week 14. *p <0.01, **p <0.001. Scale bar: 50 μm. *BMMC* bone marrow-derived mononuclear cells, *SOD1* Cu/Zn superoxide dismutase 1

Microglia cells in the anterior horn were stained with Iba1. In the presymptomatic treated animals, we observed an increase in the number of microglia in week 12 in the untreated compared with the wild-type animals (Fig. 4). The BMMC animals treated in the presymptomatic phase showed a similar increase (saline injected in week 9: 539.6 ± 149.1 cells/mm^2^, *n* = 7; BMMC injected in week 9: 495.5 ± 126.5 cells/mm^2^, *n* = 6; wild-type: 172.2 ± 116.7 cells/mm^2^, *n* = 6) (Fig. 4d).

The animals treated in the symptomatic phase showed a similar pattern as the presymptomatic groups: both saline and BMMC groups, at 15 weeks of age, had an increased number of microglia in the lumbar anterior horn compared with the wild-type animals (saline injected in week 14: 705.0 ± 190.0 cells/mm^2^, *n* = 8; BMMC injected in week 14: 861.3 ± 166.1 cells/mm^2^, *n* = 8) (Fig. 4g). However, we did not observe a difference between the treated and untreated groups.

At the end stage of the disease, the number of microglia increased greatly compared with the wild-type animals; however, there was no difference between the untreated animals and the BMMC-injected animals in the presymptomatic or symptomatic phase (saline injected in week 9: 877.56 ± 160.8 cells/mm^2^, *n* = 4; BMMC injected in week 9: 1016.36 ± 147.1 cells/mm^2^, *n* = 5; saline injected in week 14: 744.2 ± 323.8 cells/mm^2^, *n* = 5; BMMC injected in week 14: 824.5 ± 333.7 cells/mm^2^, *n* = 6).

### Fate of BMMC after spinal cord injection

**Fig. 5:**
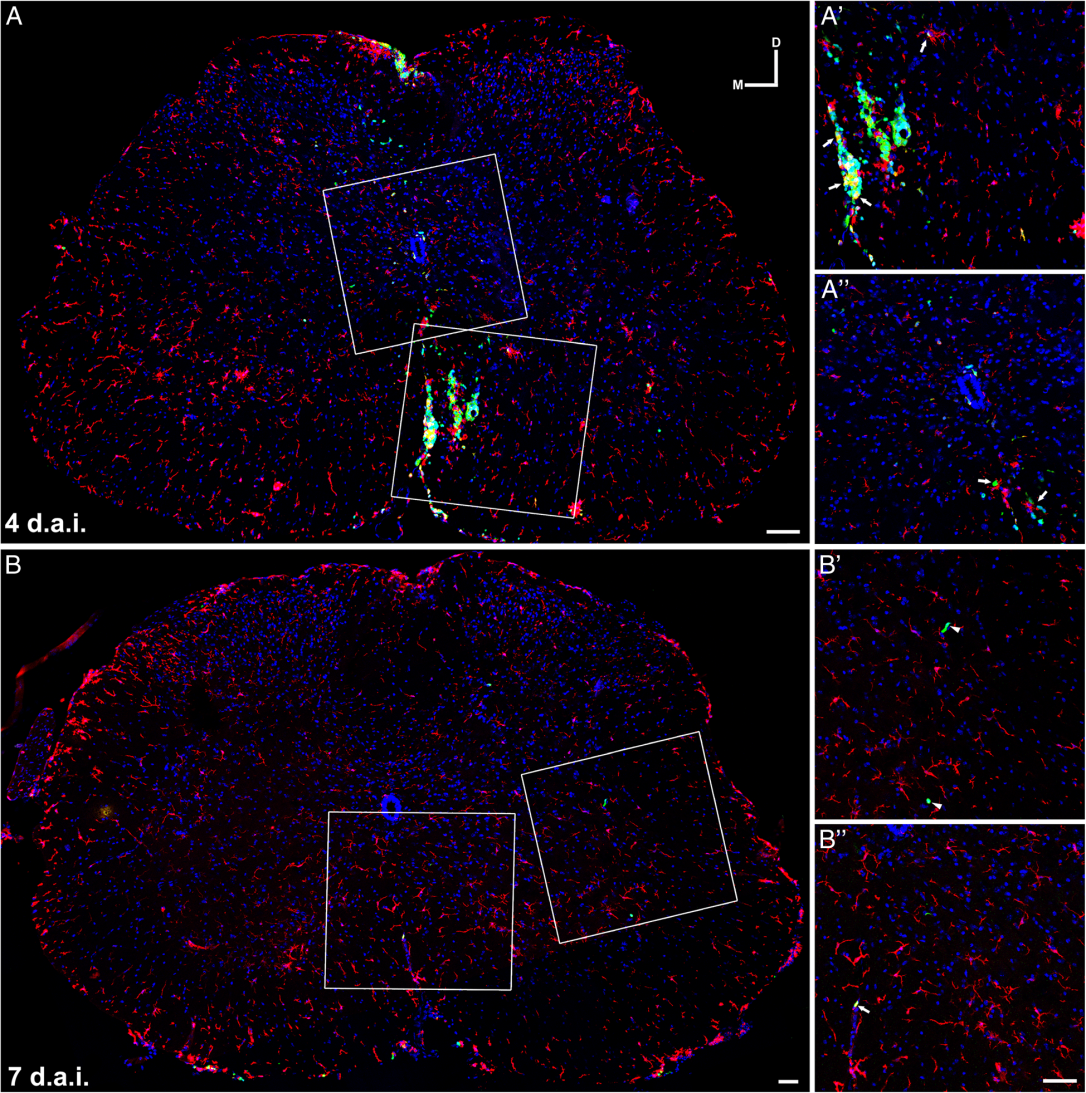
Fate tracing of BMMC-injected cells. BMMC expressing eGFP under the actin promoter were transplanted into the spinal cord of SOD1^G93A^ mice in week 9 **a, b. a** Photomontage of confocal images showing a transverse section of lumbar spinal cord 4 days after BMMC transplant. Observe the wide distribution of BMMC-eGFP^+^ (*green*) on the dorsal–ventral axis. *White boxes* in **a** show in higher magnification (**a′, a″**) the association of BMMC with microglia stained with Iba1 (*red*). *Arrows* (**a′, a″**) indicate transplanted cells associated with microglia. **b** Photomontage of confocal images showing a transverse section of lumbar spinal cord 7 days after BMMC transplant. There was a sharp decrease in the number of BMMC-eGFP^+^ in the spinal cord. Some of the few remaining cells were not clearly associated with microglia (*arrowheads* in **b′**). *Arrows* (**b″**) indicate one of the few transplanted cells associated with microglia. **b′, b″** show a higher magnification of white boxes in **b**. Scale bar: 50 μm. *d.a.i.* days after injection, *D* dorsal, *M* medial

In this study, we used different techniques in order to investigate the fate of the injected cells. In the first approach, we used cells from transgenic mice carrying the enhanced green fluorescent protein (eGFP) under the control of the actin promoter. The fate of BMMC-eGFP transplanted in presymptomatic animals was analyzed at 4, 7, and 65 (end stage) days after the injection. In sections of spinal cord tissue we observed many eGFP cells 4 days after the injection, distributed on the dorsal–ventral axis. However, the number of these cells decreased drastically by 7 days, and at the end stage we could not find any transplanted cells (Fig. 5). Analyzing the distribution of microglia (Iba1-positive cells) in the same animals, we observed that 4 days after injection many BMMC were found in association with microglia (Fig. 5a, arrows). Seven days after injection, the few BMMC observed were not clearly associated with microglia (Fig. 5b′).

**Fig. 6:**
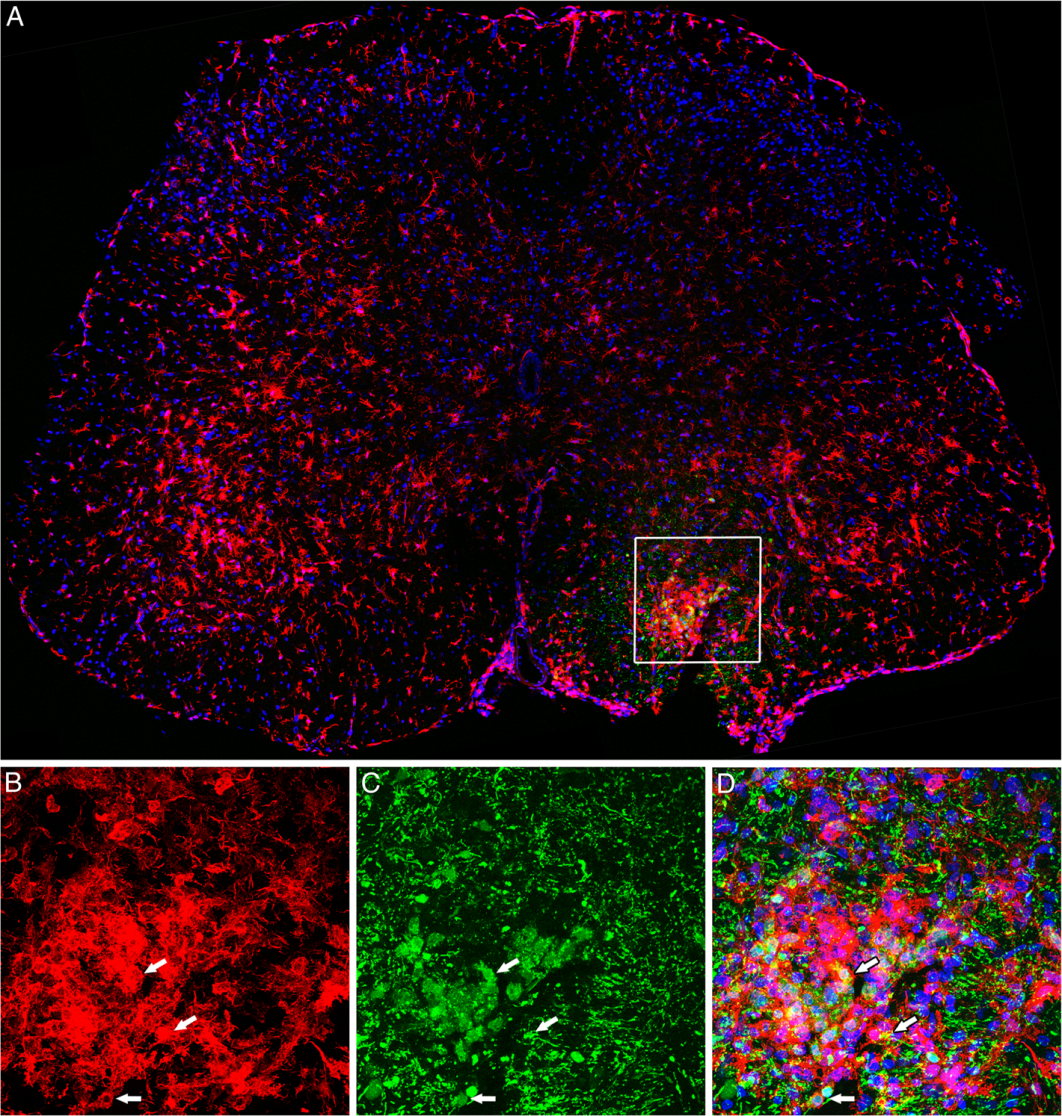
Fate tracing of BMMC-injected cells stained with CellTrace™. BMMC stained with the fluorescent cell marker CellTrace™ (*green*) were transplanted into the spinal cord of SOD1^G93A^ mice in week 14. Photomontage of confocal images showing a transverse section of lumbar spinal cord 7 days after BMMC transplant. We observed a dispersed pattern of CellTrace™ staining in the ventral horn. Microglia were stained with Iba1 (*red*). Observe the high concentration of microglia at the injection site (*white box* in **a**). **b–d** Higher magnification of the area delimited by the white box in **a**, showing the association of BMMC with microglia stained with Iba1. *Arrows* in **b–d** indicate microglia phagocyte profiles interacting with the transplanted cells. Scale bar: 50 μm

Alternatively, we also labeled the cells with the fluorescent marker CellTrace™ before injection. Seven days after injection of BMMC in a symptomatic animal, it was possible to observe CellTrace™-labeled cells in the spinal cord sections. However, we did not observe the usual cytoplasmatic pattern of labeling, and only a few cells showed the staining associated with cell nuclei (Fig. 6). Analyzing microglia in these animals, we observed a high concentration of microglia in the injection site (square in Fig. 6a and at higher magnification in Fig. 6b–d), where many cells exhibited a round phagocyte morphology similar to the transplanted cells (Fig. 6b–d, arrows).

**Fig. 7:**
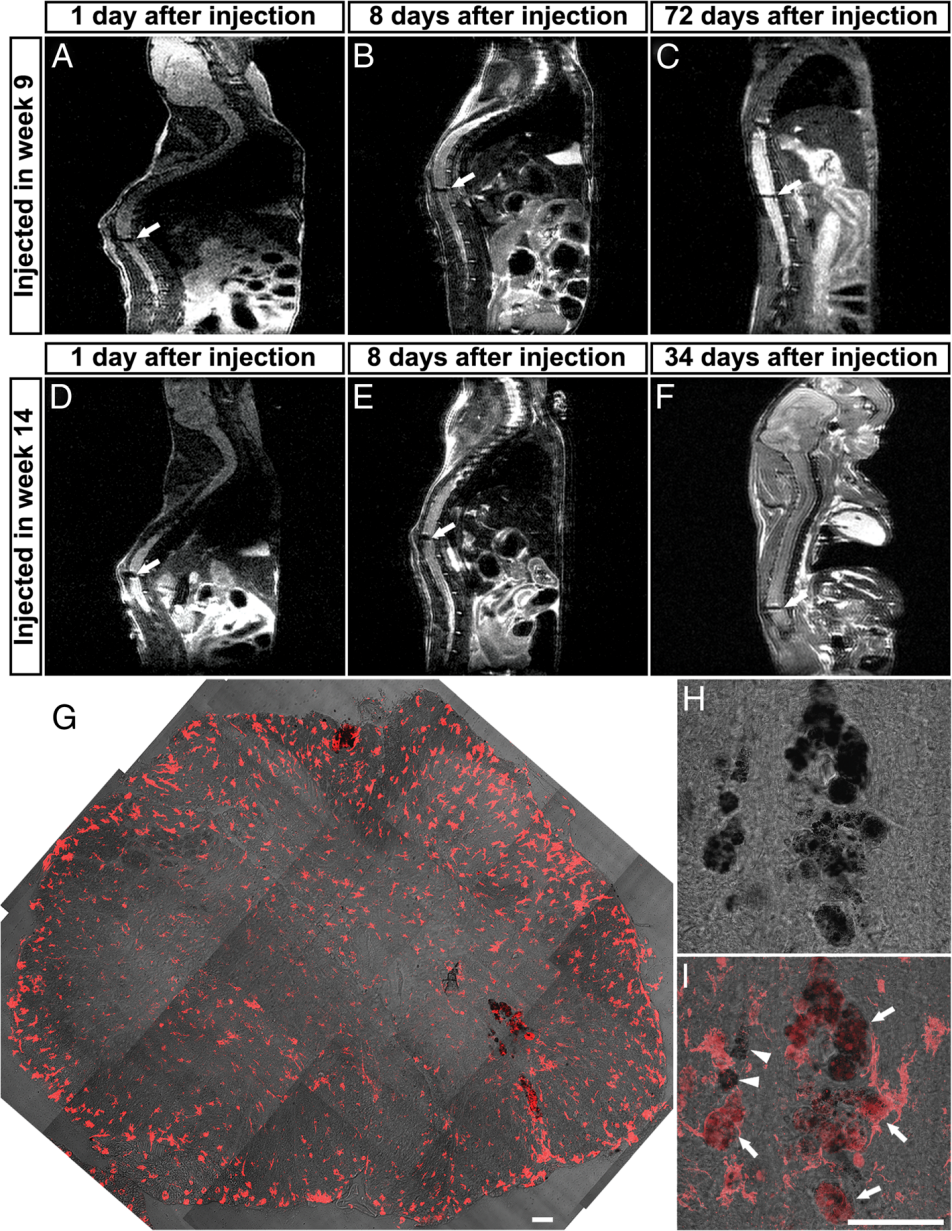
In vivo fate tracing of BMMC-injected cells. BMMC were labeled with SPION and injected into the lumbar spinal cord in week 9 **a–c** or in week 14 **d–f**. Representative images of in vivo MRI in longitudinal planes of the spinal cord at different time points. *Arrows* indicate hypointense (*black*) areas corresponding to SPION-labeled cells in the spinal cord. Labeled cells were found at day 1 **a, d**, day 8 **b, e**, and at the end stage of the disease, which corresponds to 72 days after presymptomatic injection **c** and 34 days after symptomatic injection **f. g** Transverse section of the spinal cord from the animal analyzed by MRI in **f**. SPION staining is revealed by differential and interference contrast (dark signal in **g–i**). Iba1 was used to identify microglia/macrophages (*red*). We observed only faint staining throughout the spinal cord, with most of the SPION staining located in the ventral horn. Some SPION staining was associated with Iba1-positive cells (*arrowheads* in **i**) and some with Iba1-negative cells (*arrows* in **i**). Scale bar: 50 μm

To further investigate the fate of the transplanted cells, we labeled them with SPION prior to the injection and we imaged the spinal cord region in vivo with MRI for several weeks in the same animal. We were able to detect the injected cells as a hypointense signal in the spinal cord, and this signal was present at all stages examined (Fig. 7, arrows). Importantly, even 72 days after the injection (end stage of the animal injected in week 9) the signal was still present at the injection site. Histological analyses of these animals were performed in order to identify whether the signal could be correlated with cell bodies or was due to free iron. We used differential and interferential contrast to observe the SPION and Iba1 to identify microglia/macrophages. The majority of the SPION staining was located in the ventral horn, and most of the iron seemed be associated with Iba1-positive cells (Fig. 7, arrowheads). However, it was possible to find some SPION that was not associated with the Iba1 staining (Fig. 7, arrows).

## Discussion

ALS is a fatal neurodegenerative disease for which there is no available treatment, and patients survive only 3–5 years on average after the symptoms start. ALS is a multifactorial disease, and different cell types seem to play important roles in the physiopathology. The need for an effective treatment has stimulated many researchers to test cell therapy. In our study, we injected BMMC into a mouse ALS model during the presymptomatic and symptomatic phases of the disease, to test its therapeutic potential.

Bone marrow cell therapy seems to be a promising treatment for ALS. This approach has shown good results in preclinical studies of other neurological diseases or traumas in the CNS, such as brain ischemia or optic-nerve crush [17, 25, 28]. The BMMC fraction constitutes a heterogeneous population formed mainly by hematopoietic cells, such as monocytes, lymphocytes, and granulocytes, and a small percentage of mesenchymal and hematopoietic stem cells, as described previously by our group [19]. BMMC treatment has proven to increase nerve regeneration and neural stem cell proliferation, and to reduce neuronal death and microglia activation [17, 19, 26, 29]. The main mechanism attributed to these beneficial effects is the capacity to release growth factors and cytokines [30, 31]. Besides this, the ALS model demonstrated that both wild-type monocytes and T lymphocytes (both cell types present in BMMC) delay disease progression in the mouse model, and could contribute to the beneficial effect of this therapy [4, 12].

BMMC therapy has already been tested in clinical trials for different neurological diseases [32, 33]. In ALS patients there is a clinical trial injecting these cells into the spinal cord, demonstrating that this approach is feasible and safe [23]. However, it is also important to analyze the efficacy of this therapy. Preclinical studies, as the present one, could contribute to the design of phase 2/3 clinical trials to test efficacy, such as through analysis of the best time points, dose, and routes of administration.

In this work, we performed different functional tests and histopathological analyses of the SOD1^G93A^ animals treated with BMMC in two distinctive time points. In order to analyze the functional deficit, we use three distinctive tests—rotarod, hanging wire, and motor score. Comparing the gender-matched SOD1^G93A^ animals’ performance in these tests with the wild-type animals’ performance, we could observe when the deficits start in our animals and we were able to observe a delay in the beginning of the motor loss in the BMMC-treated animals in the presymptomatic phase of the disease—although we were not able to see significant differences when we compared untreated and treated SOD1^G93A^ groups. Treatment in the symptomatic phase had no effect. Neither therapy had an effect on survival. These results are different from most of the studies that have used bone marrow cells in rodent ALS models. Nevertheless, there are important differences between our study and others that have been reported.

In mouse ALS models, wild-type bone marrow cells were used to replace the bone marrow from irradiated SOD1^G93A^ animals. The cells were transplanted in the presymptomatic phase, migrated, and invaded the spinal cord during the course of the disease. Under this approach, the results were contradictory: one group observed an increase in lifespan, while another found no positive effect [34, 35]. These conflicting results could be due to the number of cells that reached the damaged area. In our study, we injected BMMC directly into the lumbar spinal cord of presymptomatic SOD1^G93A^ mice, and we observed a positive result only in the functional outcome but not in the lifespan. However, since we performed a single local injection and we did not observe migration of these cells outside the injection site, it is possible that multiple injections are required to observe a more substantial positive effect in the functional outcome and survival. To our knowledge, this is the first report that injects BMMC into the spinal cord of SOD1^G93A^ mice.

The number of injected cells could also be crucial to obtain a positive outcome. Previous works using 10^5^ mesenchymal stem cells or 2 × 10^4^ neural stem cells observed an increase in the lifespan of ALS mice [20, 36]. Pastor et al. [37] injected 10^6^ bone marrow cells into the spinal cord parenchyma of mdf/ocd mice, and observed a beneficial functional effect. However, in the present work, using the same amount of BMMC in the SOD1^G93A^ mice model, we only saw a slight positive effect in the presymptomatic injected animals. One possible explanation is that although we injected a significant amount of cells, BMMC do not seem to persist in the spinal cord for a long period.

It is possible that the injection into the spinal cord could stimulate more inflammation, worsening the situation and leading to a decrease in the survival time. However, we also did not see a decrease in survival comparing the saline-injected animals with SOD1^G93A^ mice that did not undergo any procedure (Additional file 2: Figure S2). Moreover, considering that other groups have previously tested this approach, as already described, the strategy of injecting cells directly into the spinal cord seems to be feasible.

We observed an increase in survival in the animals treated in week 14 when compared with noninjected mice. However, we did not observe this difference between saline and treated mice in week 14. It is important to highlight that most of saline-injected and BMMC-injected mice in week 14 were littermates, which decreases possible variability. We also observed that only in the BMMC-treated mice in the symptomatic phase did a couple of animals survive more than 160 days. Thus, it is possible that by increasing the number of cells or the number of injection sites we would observe a positive effect of the therapy.

In this work, we analyzed whether BMMC therapy could affect differently male and females. It is well known that in this animal model females show a longer lifespan than males [38]. This difference could be the result of a protective role of estrogen [39]. In our study, we observe a discrete influence of gender in the functional tests. In the presymptomatic injection, the therapy seems to be more effective in females than males, since BMMC-treated female mice showed a delay in functional loss in all tests performed, while treated males showed this only in the hanging-wire test. In the symptomatic injection, when we separate the groups by gender, we were able to see an improvement or stabilization in the functional outcome, especially in males for the rotarod test. Others studies had demonstrated a distinguished response by gender in many therapies. For instance, Kondo et al. [40] demonstrated that lumbar-injected glial-rich neural progenitors improved survival only in males. Morita et al. [41], however, showed that intrathecal transplanted mesenchymal stem cells decrease disease duration only in females. So far, there is still no solid explanation for how distinguished therapies could act differently in males and females.

In this study, we tried different techniques in order to trace the fate of the injected cells. Using MRI, we could observe the BMMC injected in the same animal during the course of the disease. Until the end stage, these cells seemed to remain close to the site of injection. This low migration rate could be the reason why we observed an effect only in the functional test, but not in the lifespan, since the neurodegeneration occurs in the entire spinal cord, not only in the lumbar region. In order to overcome this possible problem, it could be interesting to test multiple injections into different regions of the spinal cord.

Importantly, we could only observe BMMC until the end stage of the disease by using SPION labeling. When we injected eGFP-BMMC or BMMC labeled with CellTrace™, we could not track the cells until the end stage. A possible explanation is that the fluorescent stain is less stable, and during the procedures to analyze the spinal cord this stain was lost. However, when we used a specific antibody against eGFP, we still could not find the cells. When we analyzed the BMMC labeled with SPION in cryosections of the spinal cord, we could see that many labeled cells were in close apposition to Iba1-positive cells. It could be argued that most of the BMMC were phagocytized by resident microglia, and these cells retained the SPION label. However, the BMMC population includes monocytes, which could survive in the spinal cord and differentiate into macrophages that also express Iba1. Nevertheless, we also found SPION-labeled cells that did not stain with Iba1, suggesting that at least a part of the BMMC population was not phagocytized.

Many groups have tried different drugs to reduce the disease progression and extend the lifespan in animal ALS models. However, although some of them showed positive effects in the animal test, the results did not translate to clinical practice [42]. One of the main concerns at present is that most of the preclinical studies treat the animals in the presymptomatic phase. In this period, the animals do not show functional deficits or significant motor neuronal death. This contrasts with the clinical trials, where the patients already show significant loss of function. In this study we injected BMMC at two different time points, in the presymptomatic phase (9 weeks old) and in the symptomatic phase (14 weeks old). We observed a positive effect in the functional tests when we injected the BMMC in the presymptomatic phase, but not in the symptomatic phase. It is possible that the environment in the symptomatic phase is already too damaged to recover with this therapy, and it is important to take this into consideration before attempting to translate this treatment to human patients.

## Conclusion

The results presented here demonstrate that BMMC therapy, used in the presymptomatic phase, could retard the loss of function in SOD1^G93A^ mice. An increase in the number of injection sites could improve this outcome. However, when BMMC were injected in the symptomatic phase we could not see any effect; this indicates that we should look for another type of cell or even combine pharmacological therapy with cell therapy to potentiate the results in the symptomatic phase.

## Additional files

Additional file 1: Figure S1. Quantification of neurons in the anterior horn of the spinal cord. The number of interneurons (NeuN-positive cells with cross-sectional area ≤250 μm^2^) was analyzed in week 12 in the presymptomatic injected animals **a** and in week 15 in the symptomatic injected animals **b**. There was no difference in the number of interneurons in the SOD1^G93A^ mice compared with the wild-type animals. The number of motor neurons was analyzed in the end stage of the disease in the presymptomatic injected animals **c** and in the symptomatic injected animals **d**. Both saline-injected and BMMC-injected mice showed a decrease in the number of motor neurons compared with the wild-type mice at the time point analyzed. ****p* <0.001. (TIF 745 kb) Additional file 2: Figure S2. Survival do SOD-1^G93A^ mice. **a, b** Comparison of survival of SOD-1^G93A^ mice that did not suffer any surgical procedure with SOD-1^G93A^ mice injected with saline at two different time points: presymptomatic phase (9 weeks old) and symptomatic phase (14 weeks old). No differences were observed between the groups. (TIF 288 kb)

## Abbreviations

ALS: Amyotrophic lateral sclerosis
ANOVA: Analysis of variance
BMMC: Bone marrow-derived mononuclear cells
CNS: Central nervous system
DMEM: Dulbecco’s modified Eagle’s medium
eGFP: Enhanced green fluorescent protein
FOV: Field of view
MRI: Magnetic resonance imaging
NGS: Normal goat serum
PBS: Phosphate-buffered saline
SEM: Standard error of the mean
SOD1: Cu/Zn superoxide dismutase 1
SPION: Superparamagnetic iron nanoparticles
