# Supplementary figures and images for: Intraspinal bone-marrow cell therapy at pre- and symptomatic phases in a mouse model of amyotrophic lateral sclerosis
Source: Stem Cell Res Ther. 2016 Mar 15;7:41. doi: 10.1186/s13287-016-0293-4 (PMC4791786; doi:10.1186/s13287-016-0293-4)

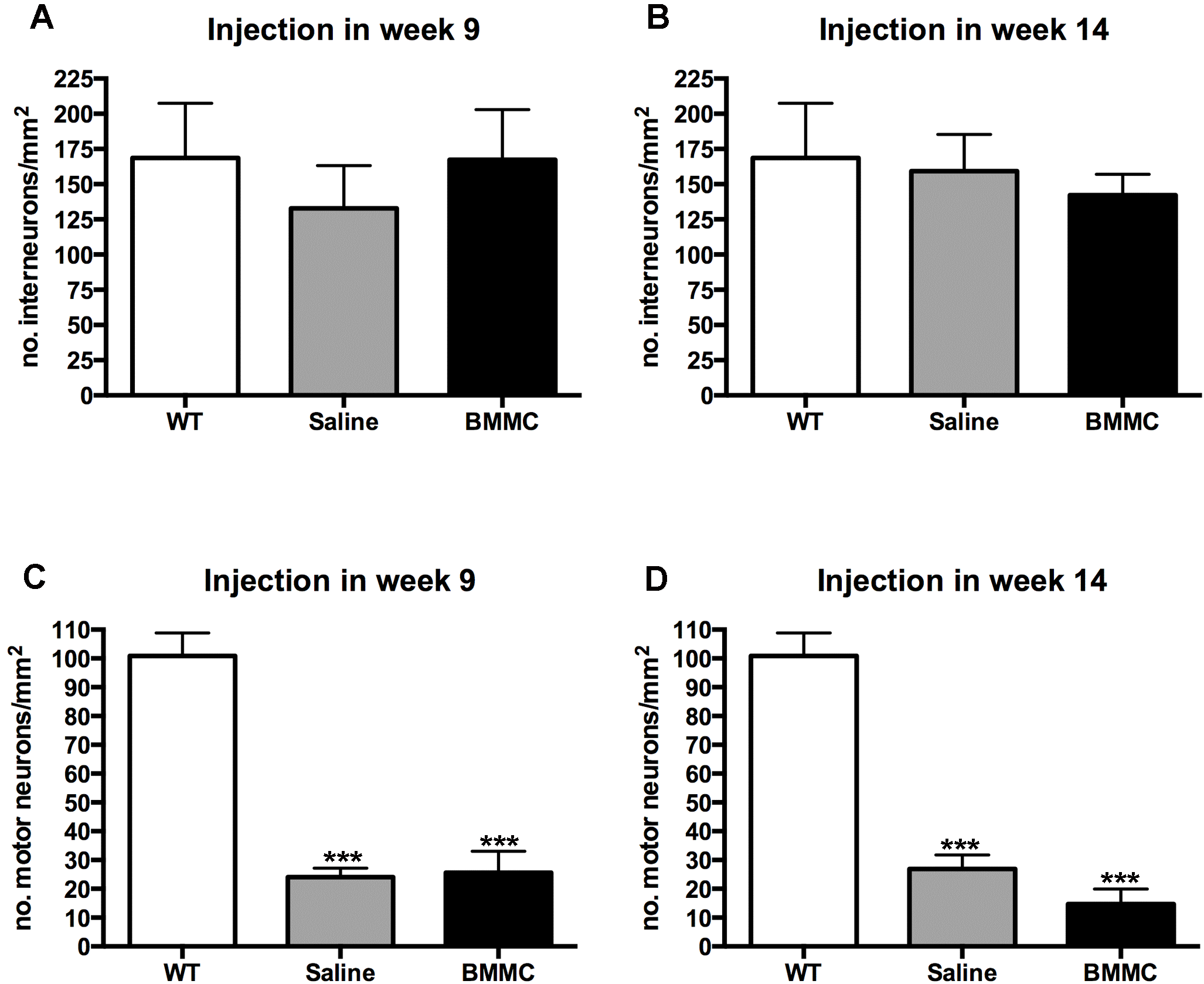

Supplement: Additional file 1: Figure S1. — Quantification of neurons in the anterior horn of the spinal cord. The number of interneurons (NeuN-positive cells with cross-sectional area ≤250 μm2) was analyzed in week 12 in the presymptomatic injected animals a and in week 15 in the symptomatic injected animals b. There was no difference in the number of interneurons in the SOD1G93A mice compared with the wild-type animals. The number of motor neurons was analyzed in the end stage of the disease in the presymptomatic injected animals c and in the symptomatic injected animals d. Both saline-injected and BMMC-injected mice showed a decrease in the number of motor neurons compared with the wild-type mice at the time point analyzed. ***p <0.001. (TIF 745 kb) [file 13287_2016_293_MOESM1_ESM.tif]

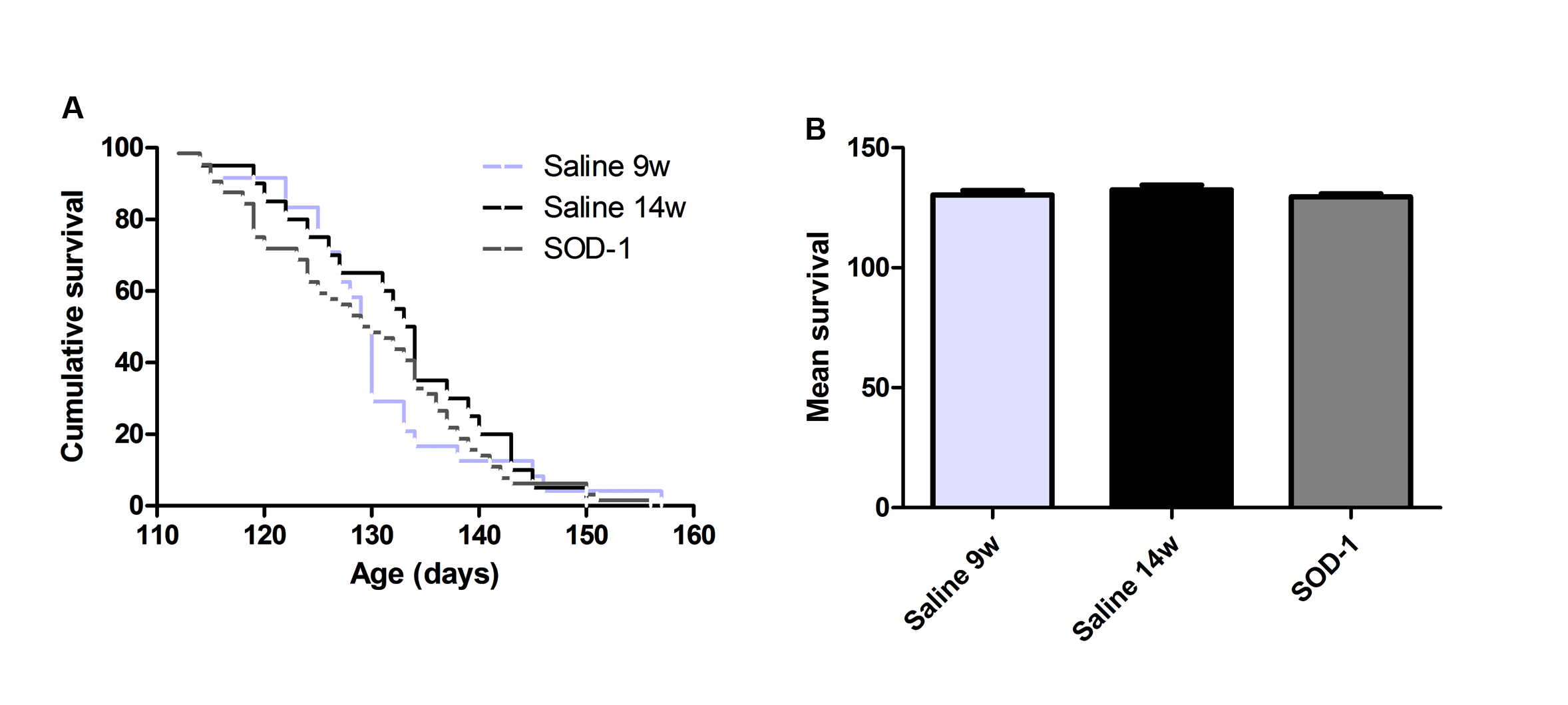

Supplement: Additional file 2: Figure S2. — Survival do SOD-1G93A mice. a, b Comparison of survival of SOD-1G93A mice that did not suffer any surgical procedure with SOD-1G93A mice injected with saline at two different time points: presymptomatic phase (9 weeks old) and symptomatic phase (14 weeks old). No differences were observed between the groups. (TIF 288 kb) [file 13287_2016_293_MOESM2_ESM.tif]
